# Reply to Graziosi et al. Rationale in the Use of Adjuvant Chemotherapy in pT3N0M0 Gastric Cancer Resected Patients. Comment on “Chen et al. Prognostic Factors and the Role of Adjuvant Chemotherapy in Pathological Node-Negative T3 Gastric Cancer. *J. Pers. Med.* 2023, *13*, 553”

**DOI:** 10.3390/jpm13060988

**Published:** 2023-06-13

**Authors:** Yi-Fu Chen, Puo-Hsien Le, Shih-Chiang Huang, Wen-Chi Chou, Jun-Te Hsu

**Affiliations:** 1 Department of General Surgery, Chang Gung Memorial Hospital at Linkou, College of Medicine, Chang Gung University, Taoyuan 33305, Taiwan; yifu061990@cgmh.org.tw; 2 Department of Gastroenterology, Chang Gung Memorial Hospital at Linkou, College of Medicine, Chang Gung University, Taoyuan 33305, Taiwan; b9005031@cgmh.org.tw; 3 Department of Pathology, Chang Gung Memorial Hospital at Linkou, College of Medicine, Chang Gung University, Taoyuan 33305, Taiwan; ab8640112@cgmh.org.tw; 4 Department of Hematology-Oncology, Chang Gung Memorial Hospital at Linkou, College of Medicine, Chang Gung University, Taoyuan 33305, Taiwan; f12986@cgmh.org.tw

We appreciate the authors very much for their interest in our article “Prognostic factors and the role of adjuvant chemotherapy in pathological node-negative T3 gastric cancer” [1]. We also greatly agree with their comments that to identify clinical and molecular predictive and prognostic factors in patients at high risk of recurrence who could benefit from the adjuvant chemotherapy is important [2]. The patients enrolled during 1994 and 2020 in our study did not receive neoadjuvant chemotherapy [1]. In this regard, recent randomized controlled studies recruiting resectable advanced gastric cancer have indicated that neoadjuvant chemotherapy (NAC) downstages the tumor and increases pathological complete response rate, with significantly longer progression-free survival in the NAC group [3,4]. However, the overall survival did not differ between the NAC group and the up-front surgery plus adjuvant chemotherapy group [3], which might, in part, be explained by the inadequate density of adjuvant treatments. Furthermore, overestimating disease severity based on imaging studies in the NAC setting may recruit patients with early-stage lesions who do not need chemotherapy.

Studies have shown that adjuvant chemotherapy prolonged overall survival in patients with stage II–III gastric cancer [5]. However, some patients did not undergo adjuvant chemotherapy under several considerations, including poor performance status after surgery, old age, concerns of chemotherapy-related side effects, and the limited benefits by chemotherapy. The role of chemotherapy in the survival node-negative T3 gastric cancer remains unclear. There is no global consensus about adjuvant treatment plans for the subgroup patients [6,7,8]. To fully clarify the issue, a large-scale randomized trial is needed.

Adequate lymphadenectomy is essential to confirm the nodal status and to stage the tumor correctly as a guide for postoperative plans for gastric cancer. Our previous study showed that the group with retrieval of lymph node > 25 exhibited the most favorable overall survival, in particular with stage II disease [9]. Furthermore, the lymph node ratio was the most important independent prognostic factor in the group with retrieved lymph node > 25 [9]. We have also investigated the prognostic significance of the number of retrieved (examined) lymph nodes in node-negative gastric cancer [10]. The results indicated that there was no survival benefit of retrieving ≥ 15 lymph nodes in T1 gastric cancer. In contrast, patients with T2–T4 lesions undergoing extensive lymphadenectomy with >25 retrieved lymph nodes had significantly prolonged overall survival and had lower locoregional recurrence rates than those with <25. Therefore, for node-negative T2–T4 gastric cancer patients with poor prognostic factors and <25 retrieved lymph nodes, adjuvant treatments, including chemotherapy and/or radiotherapy, should be considered to eradicate possible micrometastases and residual cancer cells in the locoregional nodes.

Theoretically, tailored treatment strategies should be adopted for gastric cancer patients in the era of precision medicine. However, the cost of genetic tests and the availability of molecular exams are the main barrier in the routine daily practice of managing gastric cancer. In the near future, with easily available facilities and convenient access to modern testing tools, we hope gastric cancer patients can be managed and/or treated precisely, based on precision medicine, to improve their outcomes and quality of life.
